# Genome Sequences of Two Copper-Resistant *Escherichia coli* Strains Isolated from Copper-Fed Pigs

**DOI:** 10.1128/genomeA.01341-14

**Published:** 2014-12-24

**Authors:** Freja L. Lüthje, Henrik Hasman, Frank M. Aarestrup, Hend A. Alwathnani, Christopher Rensing

**Affiliations:** a Department of Plant and Environmental Science, University of Copenhagen, Frederiksberg, Denmark; b National Food Institute, Technical University of Denmark, Lyngby, Denmark; c Department of Botany and Microbiology, King Saud University, Riyadh, Saudi Arabia; d Institute of Urban Environment, Chinese Academy of Sciences, Xiamen, China

## Abstract

The draft genome sequences of two copper-resistant *Escherichia coli* strains were determined. These had been isolated from copper-fed pigs and contained additional putative operons conferring copper and other metal and metalloid resistances.

## GENOME ANNOUNCEMENT

There is a growing concern that agricultural practices such as animal feedlots contribute to the increasing dissemination of antibiotic- and metal-resistance determinants (1). Previous work has identified an additional copper-resistance determinant named *pco* (for plasmid copper resistance) on a plasmid in *E. coli* strains from copper-fed pigs (2, 3).

Two strains of copper-resistant *E. coli* (77-3009-5, 77-30253-3) were isolated from copper-fed pigs as part of the Danish Integrated Antimicrobial Resistance Monitoring (DANMAP) surveillance program (4). The isolates were collected from healthy animals at or just prior to slaughter in 2003. Genomic DNA (gDNA) was purified from the isolates using the Easy-DNA extraction kit (Invitrogen) and DNA concentrations were determined using the Qubit dsDNA BR assay kit (Invitrogen). The isolates were sequenced on the MiSeq platform (Illumina). For sequencing on the MiSeq, chromosomal DNA of the isolates was used to create genomic libraries using the Nextera XT DNA sample preparation kit (Illumina, cat. no. FC-131-1024) and sequenced using version 3, 2 × 300 bp chemistry on the Illumina MiSeq platform.

*E. coli* strain 77-3009-5 had an estimated 5,332,861 bp on 375 contigs with the longest contig being 194,538 bp and the smallest being 94 bp. The coverage was 63-fold, and the *N*_50_ was 79,648 bp. The GC content was 50.4% and a total of 5,279 coding sequences were predicted using the Rapid Annotation using Subsystem Technology (RAST) server. *E. coli* strain 77-30253-3 was approximately 5,369,161 bp on 790 contigs. The longest contig was 211,389 bp, the smallest 101, and the GC content was 50.7%. It had an *N*_50_ of 58,415 bp; 5,294 coding sequences were predicted by RAST; and the coverage was 130-fold.

Both *E. coli* strains have genomes substantially larger than the wild-type strain *E. coli* K12 (app. 4,600,000 bp). Both strains contain the previously characterized chromosomal genes encoding proteins involved in copper homeostasis (5). In addition, *E. coli* 77-3009-5 contains a copper-resistance island with nearby Tn7-related genes. This mobile island contains two determinants: the *pco* determinant, known to give additional copper resistance (6, 7), and the *sil* determinant, which was previously shown to confer silver resistance (8). This 20-gene island is conserved in many strains of pathogenic *E. coli*, *Salmonella enterica*, *Klebsiella pneumoniae*, and *Enterobacter cloacae* (9). The genes were in the following order: *pcoE-pcoS-pcoR-pcoD-pcoC-pcoB-pcoA-pcoE-endopeptidase-hypothetical protein-silP-copG-silA-silB-silF-silC-silR-silS-silE-putative exported protein*. *E. coli* 77-3009-5 also carried an operon conferring tellurium resistance (10) and another conferring mercury resistance (11). These functions were most likely encoded on Inc-F plasmids in *E. coli* 77-3009-5, based on the presence of the different *tra* genes. *E. coli* 77-30253-3 lacks the 20-gene copper resistance island but instead has genes responsible for synthesis and handling of yersiniabactin that protects against copper toxicity (12).

### Nucleotide sequence accession numbers.

The genomes of *E. coli* 77-3009-5 and 77-30253-3 were deposited at NCBI GenBank under the accession numbers JRPP00000000 and JRQF00000000, respectively. The versions described in this paper are versions JRPP01000000 and JRQF01000000.
